# *TSC1* and *TSC2* gene mutations and their implications for treatment in Tuberous Sclerosis Complex: a review

**DOI:** 10.1590/1678-4685-GMB-2015-0321

**Published:** 2017-02-20

**Authors:** Clévia Rosset, Cristina Brinckmann Oliveira Netto, Patricia Ashton-Prolla

**Affiliations:** 1 Laboratório de Medicina Genômica, Centro de Pesquisa Experimental. Hospital de Clínicas de Porto Alegre (HCPA), Porto Alegre, RS, Brazil; 2 Programa de Pós-Graduação em Genética e Biologia Molecular, Universidade Federal do Rio Grande do Sul (UFRGS), Porto Alegre, RS, Brazil; 3 Serviço de Genética Médica, Hospital de Clínicas de Porto Alegre (HCPA), Porto Alegre, RS, Brazil; 4 Departamento de Genética, Universidade Federal do Rio Grande do Sul (UFRGS), Porto Alegre, RS, Brazil

**Keywords:** Tuberous sclerosis complex, *TSC* mutations, genotype-phenotype correlations, TSC1, TSC2

## Abstract

Tuberous sclerosis complex is an autosomal dominant disorder characterized by skin manifestations and formation of multiple tumors in different organs, mainly in the central nervous system. Tuberous sclerosis is caused by the mutation of one of two tumor suppressor genes, *TSC1* or *TSC2.* Currently, the development of novel techniques and great advances in high-throughput genetic analysis made mutation screening of the *TSC1* and *TSC2* genes more widely available. Extensive studies of the *TSC1* and *TSC2* genes in patients with TSC worldwide have revealed a wide spectrum of mutations. Consequently, the discovery of the underlying genetic defects in *TSC* has furthered our understanding of this complex genetic disorder, and genotype-phenotype correlations are becoming possible, although there are still only a few clearly established correlations. This review focuses on the main symptoms and genetic alterations described in TSC patients from 13 countries in three continents, as well as on genotype-phenotype correlations established to date. The determination of genotype-phenotype correlations may contribute to the establishment of successful personalized treatment for TSC.

## Tuberous Sclerosis Complex

Tuberous sclerosis, also known as Tuberous sclerosis complex (TSC) is an autosomal dominant neurocutaneous and progressive disorder, frequently characterized by the occurrence of multiple tumors in different organs. Penetrance reaches 95% and is variable; expressivity also varies greatly even within a given family (Northrup *et al.*, 1993). The incidence of TSC is 1/10,000 births, and its prevalence in the general population of Europe has been estimated to be 8.8/100,000 (Orphanet: Tuberous Sclerosis), affecting multiple ethnic groups (Joinson *et al.*, 2003).

### Diagnosis and symptomatology

Tuberous sclerosis has been initially described by von Recklinghausen in 1862. In 1908, Heinrich Vogt established the diagnostic criteria for TSC as the so-called triad: epilepsy, mental retardation and adenoma sebaceum. As none of these clinical signs were pathognomonic for TSC, clinical diagnostic criteria were revised by a consortium in 1998 (Roach *et al.*, 1998), which proposed three diagnostic categories (definite, probable or possible TSC) based on the presence of major and/or minor features of the disease. Table 1 shows the revised and updated diagnostic criteria for TSC, established by the same consortium in 2012 (Northrup *et al.*, 2013). A definite clinical diagnosis is made when two major features, or one major feature plus two minor features are present. Importantly, most major features are localized to the skin and central nervous system. Also, one must consider that the clinical manifestations of TSC appear at distinct developmental points, and a person with suspected TSC may need multiple sequential evaluations before a definite clinical diagnosis can be made.

**Table 1:** Revised Diagnostic Criteria for Tuberous Sclerosis Complex*.

Major Features
1. Facial angiofibromas or forehead plaque
2. Non-traumatic ungual or periungual fibroma
3. Hypomelanotic macules (more than three)
4. Shagreen patch (connective tissue nevus)
5. Multiple retinal nodular hamartomas
6. Cortical tuber^a^
7. Subependymal nodule
8. Subependymal giant cell astrocytoma
9. Cardiac rhabdomyoma single or multiple
10. Lymphangiomyomatosis^b^
11. Renal angiomyolipoma^b^
Minor Features
1. Multiple randomly distributed pits in dental enamel
2. Hamartomatous rectal polyps^c^
3. Bone cysts^d^
4. Cerebral white matter migration lines a d e
5. Gingival fibromas
6. Non-renal hamartoma^c^
7. Retinal achromic patch
8. “Confetti” skin lesions
9. Multiple renal cysts^c^
Definite TSC: Either 2 major features or 1 major feature with 2 minor features
Probable TSC: One major feature and one minor feature
Possible TSC: Either 1 major feature or 2 or more minor features

* Revised Diagnostic Criteria for Tuberous Sclerosis Complex established by a consortium in 2012 (Northrup *et al.*, 2012).

a When cerebral cortical dysplasia and cerebral white matter migration tracts occur together, they should be counted as one rather than two features of TSC.

b When both lymphangiomyomatosis and renal angiomyolipomas are present, other features of TSC should be present before a definitive diagnosis is assigned.

c Histologic confirmation is suggested.

d Radiographic confirmation is sufficient.

e One panel member recommended three or more radial migration lines constitute a major feature.

After skin and CNS findings, renal manifestations are the most common abnormalities associated with TSC. These include renal cell carcinoma, oncocytomas, angiomyolipomas (in 80% of patients) and renal cystic disease (in 50% of the patients) (Dixon *et al.*, 2011). Typically, renal manifestations in children with TSC are first seen in infancy and increase with age. Angiomyolipomas, one of the leading causes of death in TSC patients, are multiple and often bilateral. The associated mortality is due to complications when these lesions become very large. Another consequence of angiomyolipomas is destruction of the normal renal parenchyma, resulting in renal failure and end-stage renal disease (Shepherd *et al.*, 1991). Patients with clinically detectable renal cystic disease usually have a severe very early-onset polycystic phenotype (about 2% of TSC patients) (Sampson *et al.*, 1997).

Pulmonary involvement, specifically lymphangioleiomyomatosis (LAM), is the third most common cause of TSC-associated morbidity, occurring in approximately 35% of female TSC patients. LAM is caused by proliferation of atypical smooth muscle cells in the peribronchial, perivascular, and perilymphatic tissues of the lung (Kumasaka *et al.*, 2004). LAM occurs almost exclusively in young women, typically presenting between 30 to 35 years of age. Symptoms have been reported to begin or worsen during pregnancy, suggesting that LAM may be hormonally influenced (Castro *et al.*, 1995).

Skin lesions are detected in 70% of patients with TSC and include hypomelanotic macules, shagreen patches, confetti-like lesions, forehead fibrous plaque, facial angiofibromas, and periungual and ungual fibromas (Schwartz *et al.*, 2007). Depending on studied population, even as many as 100% of TSC patients younger than five years may present with hypopigmented macules. An aggregation of reddish papules, appearing on the nose and cheeks in a characteristic butterfly distribution, belongs to the Vogt triad of signs. Although usually symmetrical, occasionally they may be found unilaterally (Jozwiak *et al.*, 1998). Facial angiofibromas (adenoma sebaceum) are formed by hamartomatous growth of dermal connective tissue with rich vasculature and can result in decreased quality of life since they affect appearance, may cause disfigurement, and are prone to bleeding, which increases the possibility of infection (Yates, 2006). Shagreen Patches are areas of thick, irregularly shaped, and elevated skin, usually found on the lower back. Mean age of appearance is about 8.1 years (Sun *et al.*, 2005). Ungual and subungual fibromas are small tumors that grow around and under toenails or fingernails. Their mean age of appearance is 14.9 years (Sun *et al.*, 2005) and their prevalence in older patients (above 30 years) is close to 90%. Forehead plaques appear under the age of 14 years (Jozwiak *et al.*, 1998), with mean age of appearance being 2.6 years (Sun *et al.*, 2005).

TSC is also associated with both retinal and nonretinal ocular findings (Rowley *et al.*, 2001). Hamartomas are the most common retinal manifestation of TSC and are identified in approximately 40 to 50% of individuals. Fortunately, they rarely compromise vision, although severe decreases in visual acuity and blindness has been reported in some cases due to hamartoma enlargement, macular involvement, retinal detachment, and vitreous hemorrhage (Robertson, 1999).

Multiple cardiac rhabdomyomas are cardiac tumors most frequently encountered during infancy and childhood and they occur in approximately 30% of TSC patients. On the other side, nearly 100% of fetuses with multiple rhabdomyomas have TSC. Cardiac rhabdomyomas usually do not cause symptoms or hemodynamic compromise, and the natural history for these lesions is spontaneous regression in the vast majority of cases. However, a minority of the cases may become symptomatic shortly after birth or in the first year of life. Finally, hamartomas may also occur in organs of the endocrine system and rare case reports exist of angiomyolipomas or fibroadenomas in the pituitary gland, pancreas, or gonads (O’Callaghan and Osborne, 2010).

## Neurological involvement

Neurologic complications are the most common and often the most impairing aspect of TSC. Structural neurological abnormalities include cortical tubers, subependymal nodules (SENs) and subependymal giant cell tumors (SGCTs). Brain tumors in TSC are rare (2 to 10% of patients with TSC and 1.1-1.4% of all pediatric brain tumors) (Frèrebeau *et al.*, 1985). Cortical tubers are developmental abnormalities present in more than 88% of children with TSC (Cuccia *et al.*, 2003), and the average number of tubers per patient ranges from 5 to 50 in different studies. Tubers lead to loss of the classical six-layered cyto-architecture of the cerebral cortex and are thought to be responsible for more than 75% of the epileptic disorders in patients with TSC (Orphanet: Tuberous Sclerosis). The second more frequent structural neurological lesions in children with TSC are SENs, which are small hamartomas that occur in the walls of the lateral ventricles. Only SENs located in the region of the Monro foramina may have the potentiality to grow and to transform into SGCTs (5%-20% of patients). The last but not least important type of encephalic lesion is SGCT, affecting an average of 10% of children with TSC. SGCTs are benign, slow-growing tumors of mixed glioneuronal cells including giant cells. They are typically located near the foramen of Monro, hence they can cause increased intracranial pressure, obstructive hydrocephalus, focal neurologic deficits and death (Orphanet: Tuberous Sclerosis, 2015). Approximately 90% of TSC patients experience seizures and about 50% have documented cognitive impairment, autism, or other behavioral disorders.

Epilepsy is likely the most prevalent and challenging clinical manifestation of TSC, and virtually all subtypes of seizure have been reported. At least one third of patients develop refractory epilepsy; attention deficit-hyperactivity disorder and psychiatric comorbidities, such as mood disorders, anxiety, obsessive compulsive behavior and alcoholism are also frequently present. Among the different sites of tumor development, the brain remains undoubtedly the most problematic in terms of therapeutic management and screening. Brain tumors are the cause of more than 50% of deaths among children with TSC (Webb *et al.*, 1996). Intellectual disability has a prevalence of 40%-50% in TSC; 30% are severely affected with IQs in the very low range, and 70% have IQs in the normal, yet slightly left-shifted range (Joinson *et al.*, 2003).

### Molecular genetics of TSC: the *TSC1* and *TSC2* genes

Tuberous sclerosis is caused by mutations in one of two tumor suppressor genes: *TSC1* (9q34) and *TSC2* (16p13.3). The *TSC1* gene spans about 53kb of genomic DNA with 23 exons coding for hamartin, a hydrophilic protein with 1164 amino acids and 130 kDa. Hamartin is expressed in several adult tissues and plays a key role in the regulation of cell adhesion. This protein shows no homology with any other vertebrate protein. The *TSC2* gene comprises approximately 43kb of genomic DNA with 41 exons encoding a 5.5 kb transcript and a 1807 amino acid protein, tuberin, with 198 kDa. This protein contains a hydrophilic N-terminal domain and a conserved 163 amino acid region encoded by exons 34-38, near the C-terminal portion, which has homology with the Ras superfamily GTPases proteins rap1GAP and mSpa1 (Maheshwar *et al.*, 1997). Therefore, tuberin is a GTPase activating protein that regulates the GTP binding and hydrolysing activity of the Ras superfamily of proteins and helps to regulate cell growth, proliferation and differentiation. The other domains of tuberin are less conserved, and additional homologies between tuberin and other proteins have not been identified. Serfontein *et al.* (2011) used bioinformatics tools to examine the presence of conserved elements of *TSC1* and *TSC2* across different organisms. The analyzed organisms showed a wide range in the degree to which residues implicated in signalling are conserved (or present at all) in comparison to the human *TSC1* and *TSC2* sequences. Not surprisingly, the mammalian proteins of *Rattus norvegicus* and *Mus musculus* shared the largest number of residues with the human proteins.

**Figure 1:**
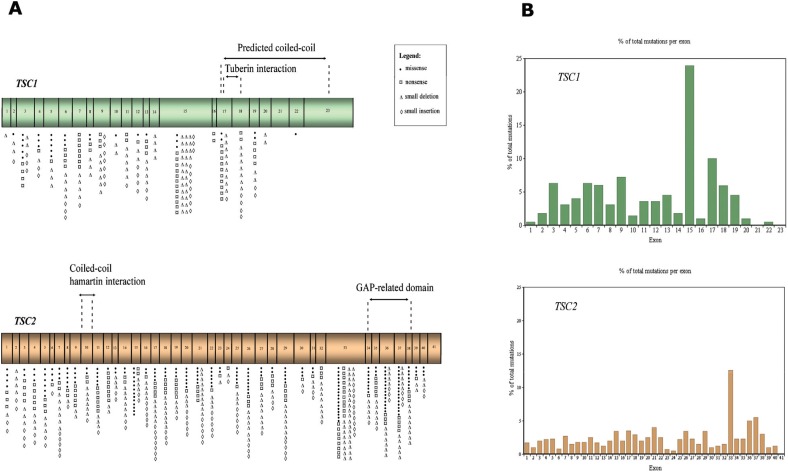
*TSC1* and T*SC2* gene structure, domains and distribution of point mutations. (A) Schematic representation of *TSC1* and *TSC2* exons and the domains of hamartin and tuberin, respectively, codified by them. The symbols represent the number of different mutations described at each exon. (B) The graph shows the percentage of the total number of described mutations that occur at each *TSC1* and *TSC2* exon.

Figure 1A schematically shows the structure of *TSC1* and *TSC2* genes, their coding exons and the main domains of hamartin and tuberin. These proteins bind each other via their respective coiled-coil domains to form an intracellular complex that integrates signals to control cellular homeostasis, oxygen levels, presence of nutrients, energy pool, and stimulation by growth factors. Such signals regulate Rheb (a Ras homologue enriched in brain), responsible for the activation of mTOR (mammalian target of rapamycin) kinase. mTOR, in turn, regulates the translation of a significant proportion of cellular proteins, including those responsible for the control of cell growth and proliferation (Kwiatkowski, 2003).

**Figure 2:**
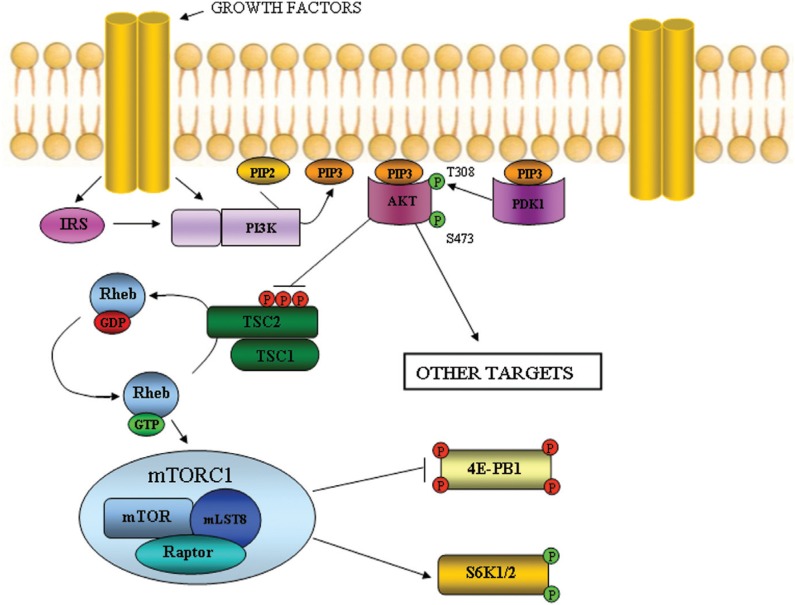
The role of the TSC2:TSC1 complex in the mTOR pathway. PI3K is activated by growth factors through direct interaction with receptors or through interaction with scaffolding adaptors, such as the IRS proteins. These interactions recruit PI3K to its substrate PtdIns(4,5)*P*2 (PIP2), allowing generation of the lipid second messenger PtdIns(3,4,5)*P*3 (PIP3). Akt and PDK1 are recruited to the cell plasma membrane through association with PIP3. This allows Akt to be activated through phosphorylation on Thr308 by PDK1 and Ser473 by mTORC2 (not shown). Once active, Akt phosphorylates many downstream targets, including multiple sites on TSC2. Phosphorylation of TSC2 impairs the GTPase activity of the TSC2:TSC1 complex, allowing Rheb-GTP to accumulate. Rheb-GTP in excess activates high levels of mTORC1, which in turn phosphorylates and inhibits 4E-BP1 and activates S6K1 and S6K2. By this way, mTORC1 influences on cell growth, translation factors activation and cell nutrition.

Figure 2 shows the role of the TSC2:TSC1 complex in the mTOR pathway. Loss of function mutations in *TSC1* or *TSC2* lead to deregulated expression patterns in this pathway, abnormal production of the end products, and ultimately promote tumorigenesis. To date, specific mechanisms by which these loss of function mutations cause disease are not established. It is suggested that tumor formation is initiated as a consequence of at least two hits (Knudson, 1971): as *TSC1* and *TSC2* are tumor suppressor genes, the inactivation of both *TSC1* or both *TSC2* alleles is necessary for benign or malignant tumor formation. The first hit is an inherited germline mutation in *TSC1* or *TSC2,* which can be detected in approximately 85% of patients with the clinical features of TSC, and the second hit is somatic. There are multiple possible mechanisms for somatic inactivation of the wild-type alleles of *TSC1* and *TSC2*, including loss of heterozygosity, mutation and promoter methylation. It is possible that epigenetic silencing mediated by micro-RNAs also occurs. Moreover, binding of TSC1 to TSC2 appears to stabilize intracellular TSC2 levels since uncomplexed TSC2 is subject to ubiquitin-mediated degradation (Chong-Kopera *et al.*, 2006). Thus, TSC1 has a role in stabilizing the complex, while TSC2 has the GTPase activity. For this reason, inactivating mutations in either gene give rise to the same clinical disorder. Clearly, both proteins play pivotal roles in several processes that are crucial for normal brain development. In addition, because they are widely expressed throughout the mature brain, these proteins likely have important homeostatic regulatory functions in neurons during adult life.

Although several TSC families exhibit an autosomal dominant pattern of inheritance, 70% of the cases result from *de novo* germline mutations. Linkage studies initially suggested that there would be equivalent numbers of families with mutations in each *TSC* gene (Benvenuto *et al.*, 2000). However, the frequency of mutations reported in *TSC2* is consistently higher than in *TSC1; TSC1* mutations account for only 10% to 30% of the families identified with TSC. In sporadic TSC, there is an even greater excess of mutations in *TSC2.* Nonetheless, identification of *TSC1* mutations appears to be twice as likely in familial cases as in sporadic cases. The disparity in mutational frequency may reflect an increased rate of germline and somatic mutations in *TSC2* as compared with *TSC1,* as well as an ascertainment bias, since mutations in *TSC2* are associated with more severe disease (Dabora *et al.*, 2001; Jansen *et al.*, 2008; Kothare *et al.*, 2014). In patients with the TSC phenotype and no identifiable mutations in either *TSC1* or *TSC2* (15% to 20%), the disease is usually milder (Dabora *et al.*, 2001). A milder phenotype has also been described in rare individuals with mosaicism for mutations in *TSC1* or *TSC2*. Caignec *et al.* (2009) reported a unique family with three independent pathogenic mutations in *TSC2* mapping to distinct haplotypes. The three mutations were most likely *de novo*, as parents of the affected patients did not present any features of TSC. In addition, findings consistent with gonadal mosaicism were seen in one branch of the family.

### Molecular diagnosis in TSC

The development of novel techniques and great advances in high-throughput genetic analysis in the last few years made mutation screening of the *TSC1* and *TSC2* genes feasible. Recent massively parallel sequencing technologies (Next-Generation Sequencing, NGS) and copy number variation testing (Multiplex Ligation-dependent Probe Amplification - MLPA and array-Comparative Genomic Hybridization - aCGH) have been validated for clinical use in many disorders including TSC, rendering the analysis much faster and more cost-effective.

Extensive studies of the *TSC1* and *TSC2* genes in patients with TSC have revealed a wide spectrum of mutations. We searched the PubMed database to retrieve available published literature in English from 1998 to 2014 that described mutations at *TSC1* and *TSC2* genes and established genotype-phenotype correlations for tuberous sclerosis disease. The following keywords were used: *TSC1* mutations; *TSC2* mutations; tuberous sclerosis complex; *TSC* mutations; *TSC* molecular analysis; genotype-phenotype correlation on tuberous sclerosis. Twenty-seven studies were included in the final analysis. Table 2 summarizes the results obtained in the main studies performed with unrelated TSC patients worldwide; many of the changes listed were found for the first time in the investigated population. The most frequent mutation type is point mutations. Large gene rearrangements are less frequently reported, both because of their true prevalence in TSC and also because several studies did not use methodologies that are directed to the identification of such mutations. As expected, the observed mutation detection rate is not always complete. In this group, a mutation could exist in an intronic region distant from the exon-intron boundaries, which could have an effect on the splicing process or gene regulation, causing a reduction of normal mRNA transcript. Although a third gene for TSC may exist and explain this lack of mutation at *TSC1* and *TSC2* genes in some patients, there is currently no concrete evidence for this. Also, somatic and germ line mosaicism is a credible explanation for the failure to detect mutations in some patients, and specialized methods can be used to enhance detection of these specific situations. Most studies in TSC patients were conducted in Europe and Asia. The largest cohorts are from the Netherlands and Poland/USA. As expected through observed mutation frequencies, in all populations described, the germline mutation rate at the *TSC2* locus was higher than that at the *TSC1* locus. Also, the frequency of small rearrangements (small insertions/deletions) is higher than missense, nonsense and splice site mutations in all populations.

**Table 2:** Type and frequency of mutations found in *TSC* genes in patients from different studies in the world and diagnostic strategy (1998-2014).

Population	N	Noncoding/polymorphic alterations	No mutation detected (%)	*TSC1*				*TSC2*				Mutation detection methods (Reference of the study)
				Point mutations (missense/nonsense)	Rearrangements		Splice site mutations	Point mutations (missense/nonsense)	Rearrangements		Splice site mutations	
					Small	Large			Small	Large		
**Europe**												
Germany	37	9	3 (8 1)	3/4	3	NA	1	10/3	8	NA	2	SSCP/Sequencing (Gumbinger *et al.* 2009)
Turkey	33	9	27 (81)	NA	NA	NA	NA	3/0	2	NA	1	SSCP/Sequencing (Apak *et al*. 2003)
Poland/USA	224	NA	38 (17)	0/11	15	0	2	31/37	43	20	27	DHPLC/Sequencing; long-range PCR/qPCR (Dabora *et al*. 2001)
Netherlands	490	76	128 (26)	0/37	38	0	7	56/67	94	20	43	SSCP/Sequencing/ Southern blot/ FISH (Sancak *et al*. 2005)
Germany	68	14	37 (54)	0/1	1	0	0	12/4	11	2	4	SSCP/Sequencing/ Southern blot/ FISH (Langkau *et al*. 2002)
Denmark	65	24	11 (17)	0/4	6	0	1	11/9	13	4	6	DGGE/ Sequencing; lomg range PCR/MLPA (Rendtorff *et al*. 2005)
United Kingdom	150	30	30 (20)	NI	NI	NI	NI	22/20	26	22	8	SSCP/ heteroduplex analysis/pulse field gel electrophoresis/ Southern blot/ long range PCR (Jones *et al*. 1999)
**SUBTOTAL**	1067	162	274 (26)	3/57	63	0	11	145/140	197	68	91	-
**Asia**												
India	24	10	12 (50)	0/0	1	NA	0	3/1	5	NA	2	SSCP/Sequencing (Ali *et al*. 2005)
China	2	NA	0	0/0	0	NA	0	0/0	1	NA	1	Sequencing (Mi *et al*. 2014)
Korea	44	NA	31 (70)	2/0	0	NA	0	0/4	6	NA	1	DHPLC/Sequencing (Choi *et al*. 2006)
Japan	21	2	0	0/1	8	NA	0	2/0	4	NA	3	DHPLC/Sequencing (Sasongko *et al*. 2008)
Japan	8	3	0	0/1	1	NA	0	4/0	0	NA	3	SSCP/Sequencing (Yamamotoa *et al.* 2002)
China	6	2	3 (50)	0/0	3	NA	0	NA	NA	NA	NA	Sequencing (Tian *et al*. 2013)
Malaysia	2	-	0	-	-	-	-	-	-	2	-	MLPA (Ismail *et al*. 2014)
Korea	11	NA	2 (18)	1/3	0	1	0	2/1	0	1	2	Sequencing/MLPA (Jang *et al*. 2012)
Taiwan	84	21	20 (24)	0/5	4	NA	0	12/15	21	NA	7	DHPLC/Sequencing (Hung *et al*. 2006)
China	6	3	3 (50)	0/0	0	NA	0	2/1	0	NA	0	Sequencing (You *et al*. 2013)
**SUBTOTAL**	184	31	59 (32)	3/11	16	1	0	22/21	32	3	17	-
**America**												
USA	21	11	0	0/5	12	0	0	NA	NA	NA	NA	Southern blot/ heteroduplex/SSCP (Kwiatkowska *et al*. 1998)
USA	126	47	52 (41)	0/7	7	NA	2	13/14	23	NA	8	SSCP/Sequencing (Niida *et al*. 1999)
USA	36	NA	7 (19)	0/1	1	0	2	4/6	6	4	2	Sequencing/MLPA (Qin *et al*. 2010)
**SUBTOTAL**	183	58	59 (32)	0/13	20	0	4	17/20	29	4	10	-
**TOTAL**	1434	251	392 (27)	6/81	99	1	15	184/181	258	75	118	-

N= number of patients included in the study; SSCP=Single Strand Conformation Polymorphism; DHPLC=Denaturing High-Performance Chromatography; FISH= Fluorescent *In Situ* Hybridization; DGGE=Denaturing Gradient Gel Electrophoresis; MLPA=Multiplex Ligation-Dependent Probe Amplification; NA=not analyzed in the study; NI=Not informed.

The exponential discovery rate of novel genomic alterations that cause TSC stimulated the creation and storage of genetic information in mutation databases. In the Human Genome Mutation Database (HGMD) for instance, 30 unique missense and 59 nonsense mutations in *TSC1* had been described by 2014, as well as 91 small deletions, 41 small insertions, 31 splice site mutations and 21 large rearrangements. In this database, TSC1 mutations correspond to 93% of the mutations, with the largest number of these occurring in exon 15, which is the largest in basepairs (559). Proportionally, it corresponds to a mutation frequency of 9.5% (determined as the percentage of mutations per base pairs, considering the size of each exon) and the highest mutation frequency is in exon 13 (14.3%). Considering all exons, the average frequency of observed mutations is 5.9%. Seven of the 23 exons have higher values (above 9%). Small deletions are responsible for 41% of the disease and small insertions, for 18.5%. Large rearrangements are responsible for 7% of mutations in TSC1 at this database. The predicted coiled-coil domain of hamartin corresponds to exons 17-23, where 21.9% of the mutations are localized. Exons 17-18 are responsible for interaction with tuberin, and they account for 15.9% of the point mutations described. Another database, the Leiden Open Variation Database (LOVD), reports 690 unique DNA variants for the *TSC1* gene.

Considering the *TSC2* gene, 183 unique missense and 125 nonsense mutations gene are described in the HGMD database, as well as 189 small deletions, 99 small insertions, 120 splice site mutations and 148 large rearrangements. Point mutations correspond to 82.6% of the mutations, with the largest number of these occurring in exon 33, which is the largest in basepairs (488). Proportionally, this exon corresponds to a mutation frequency of 15.45%, and the highest mutation frequency is in exon 37 (22.9%) and exon 38 (22.8%). Considering all exons, average frequency of observed mutations is 11.0%, a higher number than mutation frequency at the *TSC1* gene. Twenty-seven of the 41 exons have mutation frequency values above 9% and an overall mutation number and mutation frequency higher than that at the *TSC1* gene. Small deletions are responsible for 32% of the disease versus 41% in the *TSC1* gene, and small insertions for 17% versus 18.5% in the *TSC1* gene. Large rearrangements are not shown in the table, and are responsible for 17.4% of mutations in *TSC2*, a higher number than the frequency of large rearrangements in the *TSC1* gene.

The predicted coiled-coil domain of tuberin corresponds to exon 10 of the *TSC2* gene, where only 1.8% of small mutations are localized. Exons 34-38 encode the GAP-domain, responsible for the essential GTPase activaty, and they account for 18.1% of the point mutations described at this gene, with a high mutation frequency (95.1%). Exons 37 and 38 have shown the highest mutation frequency in the *TSC2* gene, and these mutations can have a damage effect on the protein since the GAP domain can be disrupted. In the Leiden Open Variation Database, 1925 unique DNA variants on *TSC2* gene have been reported.

Figure 1A illustrates the distribution of point mutations among all exons and domains of the *TSC1* and *TSC2* genes described in these different studies, and Figure 1B graphically represents the occurrence of point mutations in each of the *TSC1* and *TSC2* exons (percentage of the total number of described mutations in the HGMD database that occurs in each exon). This percentage is not related to exon size, but larger exons contain more mutations than smaller exons.

Because TSC can be a devastating disease, family members of affected individuals are often eager to know whether they are carriers of *TSC* mutations. Currently, with the adventure of next generation sequencing platforms, it became possible to analyze point mutations in both *TSC1* and *TSC2* genes at the same time for a lower cost; if no mutations are detected, the search for large deletions and duplications should proceed. Prenatal and preimplantation genetic tests are also becoming more widely available. The mutation status of family members has great implications on genetic counseling. Furthermore, for all clinical diagnostic criteria, patients with subclinical TSC may not be correctly diagnosed, and genetic testing is also very important for these cases.

The second International Tuberous Sclerosis Complex Consensus Conference brought together 79 experts from 14 countries to finalize diagnostic, surveillance, and management recommendations for patients with TSC (Northrup *et al.*, 2013). At this meeting, the most significant change recommended was the incorporation of genetic testing in the diagnostic criteria. Molecular testing of the *TSC1* and *TSC2* genes yields a positive mutation result for 75-90% of TSC-affected individuals categorized as having definite Clinical Diagnostic Criteria. The recommendation of the Genetics Panel was to make the identification of a pathogenic mutation in *TSC1* or *TSC2* an independent diagnostic criterion, regardless of the clinical findings. This will facilitate the diagnosis of TSC in some, particularly young individuals, allowing earlier implementation of surveillance and treatment with a potential for better clinical outcomes. *TSC1* and *TSC2* genetic variants whose functional effect is not definitely pathogenic would not be considered a major diagnostic criterion. Finally, a normal result from *TSC1* and *TSC2* testing does not exclude TSC, since a fraction of TSC patients has no mutation identified by conventional genetic testing. Nonetheless, if the mutation in an affected relative is known, testing for that mutation has very high predictive value for family members.

### Genotype-phenotype correlations in TSC

The discovery of the underlying genetic defects in *TSC* has furthered our understanding of this complex genetic disorder and genotype-phenotype correlations are becoming possible. In a retrospective study, Kothare *et al.* (2014) analysed a series of 919 TSC patients and found that carriage of a germline *TSC2* mutation was associated with SENs and SGCTs. Occurrence of tubers, however, did not differ between carriers of *TSC1* or *TSC2* mutations. In general, patients with *TSC2* mutation presented with symptoms at a younger age. Dabora *et al.* (2001) analyzed 224 TSC patients and found that seizures, average cortical tuber number and SEN are more frequent or severe in patients with *de novo TSC2* mutations than those with *TSC1* mutations. Jansen *et al.* (2008) also reported a more severe neurologic phenotype, including an earlier age of seizure onset, lower cognition index and more tubers in patients with a *TSC2* mutation as compared to those with a *TSC1* mutation. Another important correlation involves a subgroup of large genomic deletions at *TSC2* that also affect the adjacent *PKD1* gene, causing early-onset polycystic kidney disease (Osborne *et al.*, 1991).

Table 3 shows a compilation of the main genotype-phenotype correlations described to date. As expected, most *TSC2* mutations are generally associated with a more severe phenotype. Only one *TSC2* mutation, R905Q, was associated with milder disease. This mutation was found in 25 individuals from the same family, with a phenotype characterized by the complete absence of disfiguring skin lesions, intractable epilepsy, mental retardation, and severe organ involvement. So, the type and location of mutations in both *TSC1* and *TSC2* genes also have an influence in the phenotype. Hamartin and tuberin are known to bind to at least 40 additional proteins, and thus there are numerous potential and yet undefined effects of *TSC* gene mutations. Futhermore, it is likely that other events such as mosaicism, the nature and frequency of the second event of inactivation of the second allele and the modifying genes, as well as environmental effects may interfere with the phenotype, which makes it more difficult to establish clear genotype-phenotype correlations. Moreover, polymorphic and non-pathogenic variants in the *TSC1* and *TSC2* genes can act as phenotype modifiers in tuberous sclerosis, and they need to be further explored. To date, little is known about non-pathogenic variants in these genes, and phenotype modifiers in tuberous sclerosis have not been identified so far.

**Table 3:** Genotype-phenotype correlations established for TSC patients.

Population	N	Locus of DNA alteration	Amino acid change	Type of alteration	Main associated symptoms	Reference
EUA	1039	*TSC2*	-	Any type on *TSC2*	Mutations in the *TSC2* gene were more frequent than *TSC1* gene in patients with retinal findings	(Aronow *et al*., 2012)
Poland	170	*TSC2* c.5238-5255del 18pb	-	Frameshift	Epilepsy	(Rok *et al*., 2005)
USA	220	Contiguous deletion *TSC2-PKD1*	-	Large rearrangement	Arachnoid cysts and polycystic kidney disease	(Boronat *et al*., 2014)
Poland/USA	224	*TSC2*	-	Any type on *TSC2*	Seizures, mental retardation, average tuber count, subependymal nodules, renal angiomyolipomas, angiofibromas and fibrous forehead plaques were more common and severe in *TSC2* patients	(Dabora *et al*., 2001)
Netherlands	490	*TSC1*	-	Any type on *TSC1*	Shagreen patches are more frequent in patients with *TSC1* mutation	(Sancak *et al*., 2005)
Netherlands	490	*TSC2*	-	Any type on *TSC2*	Mental retardation is more frequent in patients with *TSC2* mutation	(Sancak *et al*., 2005)
Netherlands	490	*TSC2*	-	Nonsense and frameshift	Shagreen patches, forehead plaques, facial angiofibromas, ungual fibromas, renal angiomyolipomas and renal cysts	(Sancak *et al*., 2005)
Netherlands	490	*TSC2*	-	Mutations in the GAP domain	Mental retardation, seizures and subependymal nodules	(Sancak *et al*., 2005)
Korea	11	*TSC2*	-	Mutations in exons 33-41	Cardiac rhabdomyomas	(Jang *et al*., 2012)
USA	65	*TSC2*	-	Any type on *TSC2*	Higher number of cysts than *TSC1* woman with pulmonary lymphangioleiomyomatosis	(Muzykewicz *et al.*, 2009)
Canada	19 families	*TSC2*	R905Q	Missense	Milder disease severity	(Jansen *et al.*, 2006)
USA	478	*TSC2* proximal region (exons 1-22) and distal region (exons 34-41)	-	Missense mutations and small in-frame deletions or insertions	Proximal and distal *TSC2* mutations showed a significantly higher risk of infantile spasms compared with mutations in the central region of the gene	(van Eeghena *et al.*, 2013)
USA and Belgium	919	*TSC2*	-	Any type on *TSC2*	More frequent occurrence of several kinds of seizures/epilepsy subtypes: partial epilepsy, complex partial seizures, infantile spasms, SENs, SGCTs and cognitive impairment.	(Kothare *et al*., 2014)
United Kingdom	One case report	*TSC1* intron 10 (c.1030-3 C > G)	-	Splice site mutation	Mild phenotype (seizures and small number of hypomelanotic macules)	(Blyth *et al*., 2010)

N= number of patients included in the study.

NI=Not informed.

In light of emerging human genetic and molecular knowledge, molecular diagnosis of TSC and determination of genotyope-phenotype correlations might help in the establishment of personalized treatment for TSC patients and improve quality of life among these patients. Continuous studies in this area can guide future directions in this line.
